# Current Devices and Complications Related to Transcatheter Mitral Valve Replacement: The Bumpy Road to the Top

**DOI:** 10.3389/fcvm.2021.639058

**Published:** 2021-06-11

**Authors:** Faraj Kargoli, Matteo Pagnesi, Kusha Rahgozar, Ythan Goldberg, Edwin Ho, Mei Chau, Antonio Colombo, Azeem Latib

**Affiliations:** ^1^Department of Cardiology, Montefiore Medical Center/Albert Einstein College of Medicine, Bronx, NY, United States; ^2^Cardio-Thoracic-Vascular Department, San Raffaele Scientific Institute, Milan, Italy; ^3^Department of Internal Medicine, Montefiore Medical Center/Albert Einstein College of Medicine, Bronx, NY, United States; ^4^Department of Cardiothoracic Surgery, Montefiore Medical Center/Albert Einstein College of Medicine, Bronx, NY, United States; ^5^Interventional Cardiology Unit, GVM Care & Research Maria Cecilia Hospital, Cotignola, Italy

**Keywords:** TMVR, TMVR complications, lvot obstruction, valve thrombosis, valve embolization

## Abstract

Mitral regurgitation is the most common valvular lesion in the developed world, with increasing prevalence, morbidity, and mortality. The experience with surgical mitral valve repair or replacement is very well-validated. However, more than 45% of these patients get denied surgery due to an elevated risk profile and advanced disease of the left ventricle at the time of presentation, promoting the need for less invasive transcatheter options such as transcatheter repair and transcatheter mitral valve replacement (TMVR). Early available TMVR studies have shown promising results, and several dedicated devices are under clinical evaluation. However, TMVR is still in the early developmental stages and is associated with a non-negligible risk of periprocedural and post-procedural complications. In this review, we discuss the current challenges facing TMVR and the potential TMVR-related complications, offering an overview on the measures implemented to mitigate these complications, and future implications.

## Introduction

Mitral regurgitation (MR) is the most common valvular disease in the developed world and is associated with high morbidity and mortality. The prevalence of MR increases with age, reaching up to 10% in individuals over the age of 75 (1, 2). Surgical mitral valve repair (MVr) or replacement has been a very well-established therapeutic option (3, 4). However, due to the high risk associated with surgical interventions, and the level of advanced disease in this patient population, more than 49% of MR patients get rejected from surgical mitral valve replacement (5, 6). This creates an unmet clinical need and a push for novel less invasive percutaneous mitral valve treatment alternatives—whether repair or replacement—with less periprocedural mortality and good clinical outcomes (7, 8).

Transcatheter MVr is a well-established treatment strategy for MR (primary and secondary MR), with more than 100,000 transcatheter MVr cases performed to date worldwide (9). However, transcatheter MVr has technical challenges and limitations: MR reduction is not always guaranteed, suboptimal anatomies limit patient suitability, limited percutaneous options if MR recurs, a single repair device runs the risk of being inadequate, and many cases may require more than one device to achieve surgical-like repair results (10, 11). Therefore, to accommodate the unmet needs of this patient's population, transcatheter mitral valve replacement (TMVR) has emerged as a promising intervention that can help reduce MR in non-surgical patients and in those with unsuitable anatomy for transcatheter edge-to-edge repair. Moreover, TMVR represents a new treatment option for inoperable or high-risk patients with degenerated or failed bioprostheses or failed repairs [valve-in-valve (ViV) or valve-in-ring (ViR), or in patients with severe annular calcifications, valve-in-mitral annular calcification (ViMAC)] (12). Despite the technological advancements in the field of structural heart disease, TMVR is still being performed in very high-risk surgical patients, restricted to high-volume experienced centers, and with a relevant risk of periprocedural and post-procedural complications. In one study examining outcomes of 203 patients with severe MR who have been excluded from the available TMVR protocols, more than 88% of patients were rejected from the early feasibility studies of TMVR due to advanced frailty, with mortality reaching up to 12% in those who were ineligible. That same study found that frailty (15%), severe tricuspid regurgitation (TR) (14%), prior aortic valve therapy (13), mitral anatomical exclusion (16%), severe MAC (7%), and risk of left ventricular outflow tract (LVOT) obstruction (LVOTO; 4%) are the most common causes of TMVR exclusion (6). This review offers a brief insight on the current challenges, potential complications of TMVR, and the measures available to mitigate these complications.

## Challenges in Designing a Transcatheter Heart Valve for the Mitral Position

The mitral valve apparatus is a complex structure, consisting of highly dynamic annulus, the two leaflets (anterior and posterior), chordae tendinae, and papillary muscles (14). Moreover, the advanced disease of the left ventricle (LV) at the time of presentation due to different etiologies can limit the available transcatheter options and patient's eligibility. One advantage of surgical MV intervention is that it can be tailored to target the specific pathology of the valvular apparatus. However, today with the detailed pre-procedural planning and with the availability of several transcatheter devices, a targeted pathology approach could be achieved with transcatheter interventions (13). Moreover, the saddle asymmetrical shape of the non-stiff mitral valve annulus and its anatomical relation to the insertion of the papillary muscles make the task to design a transcatheter heart valve (THV) for the treatment of MR incredibly challenging. The ideal THV should match the native annulus without running the risk of delayed device detachment, paravalvular leak (PVL), or MR recurrence (15).

## Current Evidence and Early Experience

TMVR complications can be divided based on either the time of occurrence (procedural vs. post-procedural) or complications related to the device or to its pathway (16, 17). For the purpose of simplicity and due to the different risk profiles, we will divide the complications into procedural and post-procedural (Table 1), stratified by TMVR in native valve, ViV, ViR, and ViMAC. The first TMVR case series was performed in a failed prosthesis as ViV; all patients received inverted Edwards Life Sciences Sapien XT (Edwards Lifesciences, Irvine, CA) valve through the transapical (TA) approach (18). This was followed by TMVR in native valve (19). Early feasibility TMVR in native valve studies with relatively small sample sizes tested the safety and efficacy of these new THVs are summarized in Table 2 (20–28). Whereas Table 3 reports data on ViV and ViR and Table 4 reports data on VinMac procedures (16, 21, 22, 29–31, 33–43, 45–47). The early experience from early feasibility studies and registries showed that the anatomical nature and complexity of the mitral valve impose unique complications that are summarized in Table 1.

**Table 1:** TMVR complications stratified into procedural vs. post-procedural.

**Short term complications *(peri-procedural)***	**Long term complications (*post-procedural)***
Valve embolization or late migration	Severe PVL/Hemolysis
Need for second valve/Reintervention	Valve Thrombosis/Dysfunction
Damage/interference with other structures • LV perforation • LV pseudoaneurysm • Mitral annular disruption • LCx occlusion • MV leaflet/Chordal disruption • Pulmonary vein perforation	Residual moderate to severe MR
Conversion to open heart surgery	Cerebral embolic events (clinical or subclinical)
LVOT obstruction	Durability
Residual MR right after procedure	Post procedural ASD
Access related complications • Transapical • Transfemoral • Transatrial	Non TMVR related complications • Prolonged length of stay • Iatrogenic nosocomial infections

*TMVR, transcatheter mitral valve replacement; LV, left ventricle; LCx, left circumflex artery; MV, mitral valve; PVL, paravalvular leak; LVOT, left ventricular outflow tract; MR, mitral regurgitation; ASD, atrial septal defect*.

**Table 2:** Early feasibility studies in native mitral valve.

**Study**	**THV used**	**N, Follow up time**	**Mortality**	**Successful implantation**	**TVE**	**LVOTO**	**Bleeding**	**Miscellaneous**
Intrepid 20	Intrepid	50, 1 year	24%	98%	None	None	9 (18%)	Five Reintervention for bleeding. Apical bleeding was an issue
CardiaQ 21	CardiAQ valve	12, 30 day	17%	75%	Not reported	Not reported	Not reported	1 Death procedure related
Highlife 22	HighLife	15, 30 day	21%	64%	N/A	1 (7%)	N/A	1 Patient with valve thrombosis
TIARA 23	Tiara	56, 90 day	21%	95%	5% (migration)	None	5.3% access site complication	5.3% conversion to open heart surgery 2% stroke 2% MI 14% AKI
Tendyne 24	Tendyne	100, 1 year	26%	96%	4% (migration)	None	32%	4 Reintervention/device retrieval 7 Valve thrombosis 3 Endocarditis, 5 Disabling stroke 11 Pacemaker implantations 52 Vascular complication
Sapien M3 25	Sapien M3	15, 30 day	0%	93%	None	None	None	1 PVL 1 Stroke 3 Rehospitalized (device related)
Fortis 26	Fortis	13, 2 year	39%	77%	None	None	None	New onset arrhythmias 15%
PRELUDE 28	Caisson	18, 304 day	18%	92%	None	None	None	4 Converted to surgery 1 Retrieved 1 PVL 1 reintervention 1 New onset AF 2 Stroke HF rehospitalization
Modine et al. (20)	Cephea	1, 28 weeks	0%	100%	None	None	None	

*ViV, valve-in-valve; ViR, valve-in-ring; LVOTO, left ventricle outflow tract obstruction; THV, transcatheter heart valve; CVA, cerebrovascular accident; LV, left ventricle; MI, myocardial infarction; AKI, acute kidney injury; PPM, pacemaker placement; VSD, ventricular septal defect; AF, atrial fibrillation; PVL, paravalvular leak*.

**Table 3:** TMVR studies stratified by ViV and ViR.

**References**	**Study Design**	***N***	**THV used**	**Years follow up**	**Mortality**	**CVA** **(any type)**	**LVOTO**	**TVE**	**Major** **bleeding**	**THV thrombosis Dysfunction**	**LV perforation/conversion to surgery/other**
Cheung et al. (18)	Single center	23 ViV	Sapien XT Sapien	2 years	ViV 10% 4.5% CVD	4%	None	1 (4%) atrial migration	26%	4%	One PPM One PVL with reintervention
Eng et al. (29)	Multicenter retrospective	60 ViV 15 ViR	Sapien XT/3	1 year	ViV 14% ViR 18%	Not reported	ViV 5% ViR 20%	3 (6%)	ViV 7% ViR 13%	ViV 2% ViR 7%	Three required second THV Four conversion to open heart surgery One LV pseudoaneurysm
Bouleti et al. (30)	Single center prospective	34 ViV 30 ViR	Sapien XT Sapien 3	30-days	ViV 6% ViR 7%	ViV 6% ViR none	ViV (6%) ViR (13%)	sViV 1 (3) ViR 1 (3)	ViV 6% ViR 3%	ViV 9% ViR 7%	Two ViR converted to open heart Six needed second valve
MITRAL VIVID 30	Multicenter retrospective	349 ViV 88 ViR	347 Sapien XT 28 Melody 17 Sapien 3 18 miscellaneous	30-days	ViV 8% ViR 11%	ViV 3% ViR 1%			N/A	N/A	
TMVR registry 39	Multicenter retrospective	322 ViV 141 ViR	247 Sapien 3 175 Sapien/XT 21 Lotus 16 Direct Flow 4 Melody	1 year	ViV 14% ViR 31%	ViV 2% ViR none	ViV 7 (2%) ViR 7 (5%)	ViV 3 (1%) ViR 2 (1%)	ViV 7% ViR 11%	ViV 10 ViR 1	Four LV perforation 25 needed second valve 60 (13%) needed reintervention
Kamioka et al. (31)	Multicenter retrospective	62 ViV	21 Sapien XT 41 Sapien 3	1 year	ViV 11%	None	2 (3%)	None	Life threatening 7% Bleeding 16%	1 (2)	One PVL required reintervention
MITRAL trial 45	Multicenter prospective	26 ViV 30 ViR	Sapien XT/3	30 days	ViV 4% ViR 7%	None	None	None	ViR 3%	None	Six Need for second valve Four persistent MR One needed reintervention
El Sabbagh et al. (32)	Single center retrospective	14 ViV 10 ViR	16 Sapien XT 8 Sapien 3	1 year	22%	1 (4.2%)	N/A	None	Life threatening 8%	N/A	
Yoon et al. (33)		28 ViR	17 Sapien XT 10 Sapien 3	1 year	3 (13%)	2 (7%)	1 (3.6%)	1 (3.6%)	Bleeding 14%	4%	Five needed second valve Seven rehospitalized Two converted to open heart
Yoon et al. (33)	Single center retrospective	6 ViV 11 ViR	Sapien XT	18 months	32%	None	1 (1/17) migration	None	Major bleeding 6%	N/A	One PVL
Guerrero et al. (34)	Multicenter retrospective	ViR 17	Sapien XT	1 year	38%	N/A	1 (1/17)	N/A	N/A	N/A	One conversion to open heart due to TVE One THV implantation too atrial- needed a second valve
Cullen el al. (35)	Single center Retrospective	8 ViV 5 ViR	Sapien XT Sapien 3	6 months	8%	8%	None	None	Major bleeding 8%	2 (15%)	One needed second valve Four AKI One major stroke
Guerrero et al. (36)	Single center Retrospective	10 ViV 2 ViR	Sapien XT	During admission	15%	None	None	None	None	14%	One PPM implantation
Seiffert et al. (37)	Single center Case series	7 ViV 2 ViR	Sapien/XT	During admission	None	None	N/A	N/A	None	22%	Uneventful TMVR 3 valve thrombosis
Werner et al. (38)	Single center Case series	9 ViV	Melody	6 months	43%	None	None	None	N/A	None	One transseptal closure Four with vascular access site complications Two hemothorax
Kliger et al. (39)	Multicenter retrospective	8 ViR	Direct Flow	30 days	25%	None	1 (1/6) One initially then repositioned	none	N/A	None	Two LVOTO 1 was relieved and one needed alcohol septal ablation
Cerillo et al. (40)	Single center Case series	6 ViV	Sapien/XT	70 days	17%	None	None	None	1 (33%) GI bleeding	None	One major bleeding from TA wound
Werner et al. (38)	Single center Case series	5 ViV	Melody	During admission	None	N/A	1	None	N/A	None	Four out of Five successful melody implantations PVL
Descoutures et al. (41)	Single center Case series	3 ViV	Sapien	During admission	33%	N/A	None	None	N/A	None	LV pseudoaneurysm
Wilbring et al. (42)	Single center	3 ViV 1 ViR	Sapien 3	1 year	None	None	None	None	None	None	One patient with complete heart block

*TMVR, transcatheter mitral valve replacement; ViMAC, valve-in-mitral annular calcification; LVOTO, left ventricle outflow tract obstruction; THV, transcatheter heart valve; TVE, transcatheter valve embolization; CVA, cerebrovascular accident; LV, left ventricle; MI, myocardial infarction; AKI, acute kidney injury; PPM, pacemaker placement; VSD, ventricular septal defect; AF, atrial fibrillation; PVL, paravalvular leak*.

**Table 4:** 

**Study**	**Design**	**N, STS %**	**THV used**	**Years follow up**	**Mortality**	**CVA any type**	**LVOTO**	**TVE**	**Major bleeding**	**THV thrombosis dysfunction**	**LV perforation/conversion to surgery**
Guerrero et al. (16)	Multicenter retrospective	116	57 Sapien XT 57 Sapien 3 2 Inovare	1 year	54% CVD 24%	9%	11%	5 (4%)	3	2%	17 needed second valve (11 due to MR, 6 due to migration) 2 LV perforation 4 converted to open heart surgery
STS/ACC TVT Registry 46	Multicenter retrospective	100	50 Sapien 3 50 Sapien XT	30 day	22%	6%	10 (10%)	4 (4%)	Not reported	None	3 Cardiac perforation 4 needed second valve 4 vascular complications 2 conversion to open heart 3 PPM implantation
TMVR Registry 39	Multicenter retrospective	58	41 Sapien 3 9 Lotus 6 Sapien XT 2 Direct Flow	1 year	63%	2%	23 (40%)	4 (7%)	3 (5%)	None	5 converted to open heart 3 needed second heart valve 13 needed reintervention
MITRAL Trial 45	Multicenter prospective	30	Sapien XT/3	30 day	19% 4% CVD	4%	3 (10%)	None	2 (4%)	None	1 needed second THV 2 with persistent MR 1 LV perforation 1 VSD 3 Hemolysis 4 PPM implantation 1 Pericardiocentesis
Urena et al. (43)	Single center prospective	27	5 Sapien XT 22 Sapien 3	30 day	3 (11%)	2 (7%)	2 (7%)	3 (11%)	1 (4%)	3 (11%)	6 needed second THV 2 Major vascular complications
Eleid et al. (44)	Multicenter retrospective	12	Sapien XT/3	1 year	43%	N/A	17%	2 (17%)	25%	None	2 required second THV 1 required conversion to surgery 1 severe PVL
Praz et al. (22)	Multicenter retrospective	26	24 Sapien 3 2 Sapien XT	30 day	27%	1 (4%)	1 (4%)	None	2 (8%)	None	4 AKI 2 PPM 7 AF
Russell et al. (21)	Single center retrospective	8	Sapien 3	30 day	None	None	None	None	None	None	1 mild PVL/hemolysis closed with vascular plug 1 died at 7 months
Werner et al. (38)	Single center	3	Sapien 3	1 year	33%	None	None	None	None	None	1 patient suffered pneumonia and sepsis died post procedural day 12

## Complications in Transcatheter Mitral Valve Replacement

### Valve Embolization or Early/Late Migration

Prosthetic valve embolization has not been reported in the surgical literature, and it has been described as a unique complication of transcatheter valves (48). The friction between the frame of the transcatheter prosthetic mitral valve and the surrounding tissue generates the anchoring force of the THV. Therefore, deployment in suboptimal position could decrease this force, leading to malposition or migration. In the TMVR case series of 23 consecutive patients undergoing mitral ViV by Cheung et al. (18), one patient was readmitted with heart failure, and echocardiography showed 5-mm valve migration to the left atrium with severe intervalvular regurgitation that required a second uneventful TA TMVR. In the ViMAC study by Guerrero et al. (16, 49), six patients had migration of the implanted device and five patients with TVE required a second THV. Bapat et al. (50) reported two cases of device migration after successful THV implantation and delayed presentation of recurrent severe MR on echocardiography within 1 week and 3 months. Both cases were treated with open surgical mitral valve replacement. Upon further study of the explanted bioprosthesis, the authors hypothesized that delayed migration occurred due to the elevated closing pressure of the LV that the device must cope with, THV undersizing, and the lack of extensive calcification of the mitral leaflets (50).

The treatment of valve migration or embolization can be performed by transcatheter snaring, re-do transcatheter ViV, or open-heart surgery. Choosing the right option of treatment depends on the severity of MR, the urgency of treatment, the migrated valve position, and the patient's surgical risk profile (51).

### Left Ventricular Outflow Tract Obstruction

Severe LVOTO is a life-threatening complication of TMVR. The native LVOT is confined by the most basal septum, intervalvular fibrosa (aortomitral tissue), and the basal portion of the anterior mitral leaflet (AML). AML sequestration by the newly implanted THV can lead to elongation of the LVOT, determining what is known today as the neo-LVOT (25, 52). The risk of LVOTO can be predicted on pre-procedural multidetector cardiac tomography (MDCT), which can help inform the optimal depth of device implantation and the need for further intervention by predicting the neo-LVOT area. A decrease in neo-LVOT area is a risk factor for LVOTO, which can manifest as immediate hemodynamic instability after THV deployment, with intra-procedural echocardiographic evidence of valve displacement or AML sequestration leading to LVOTO, and LVOT gradient >10 mmHg than baseline (53). Factors that are taken into consideration when analyzing pre-procedural imaging are the AML length, neo-LVOT area <200 mm^2^, device-related dimensions, aortomitral angle, and basal septal bulge. Three-dimensional (3D) prototyping of available cardiac CT images can be used to predict the risk of LVOTO and enhance procedural outcome (54). In one study evaluating eight patients who underwent TMVR, when compared to post-procedural imaging, 3D printed models were able to predict LVOTO in two out of the five printed models (55, 56). In an analysis of approximately 200 cases of the TMVR international multicenter registry, the prevalence of LVOTO was 13%, with the highest rate in ViMAC, then in ViR and ViV (54, 8, and 2%, respectively). Moreover, the authors showed that the estimated neo-LVOT area (measured during mid-end systole on MDCT) was inversely related to LVOT gradient and significantly correlated with the actual neo-LVOT area after THV deployment. Other predictors of LVOTO were distance of the mitral annulus to the interventricular septum and left ventricular end diastolic diameter. Patients with LVOTO had higher rates of procedural adverse events and related deaths (35 vs. 2%, *P* < 0.001) (57). In patients with LVOTO (*N* = 26), 11% were managed medically, 19% underwent emergent open-heart surgery, 8% underwent emergent TAVR, while alcohol septal ablation (ASA) was performed in 31% of patients.

In the ViMAC registry by Guerrero et al. (16), LVOTO was associated with all-cause mortality. LVOTO happens in mid-late systole, and it is at the end of systole when the LVOT is at its smallest diameter (45% of the cardiac cycle on MDCT) (57). However, in a retrospective analysis using a novel approach of physiologic early systolic assessment of the dynamic LVOT, the authors found that measuring LVOT in end systole may overestimate the risk of LVOTO and could increase the rate of screen failure due to non-anatomical conditions. Moreover, they proposed a novel multiphase physiological evaluation of the LVOT, which leads to an increase in their TMVR patients' eligibility by more than 50%, and no cases of LVOTO at 30-days follow-up (58). These observations are indicators of the learning curve and improvement of the groundwork for the TMVR procedure and its complications. The high prevalence of this complication and its association with mortality lead to a reassessment of the steps followed in the evaluation and pre-procedural planning of TMVR. Moreover, implementation of multiple imaging modalities to help predict LVOTO and intra-procedural bailout strategies have been described (59–62). Pre-TMVR screening remains an evolving field with data from ongoing registries continuing to contribute to our understanding and learning.

## Available Techniques/Modalities to Mitigate Left Ventricular Outflow Tract Obstruction

### Transesophageal Echocardiogram

Intra-procedural transesophageal echocardiogram (TEE) with 3D imaging is key to identify the MV relationship to adjacent structures, which can help improve procedural outcomes and lower the risk of LVOTO (56).

### Surgical Management of Left Ventricular Outflow Tract Obstruction

AML laceration is a well-established treatment of systolic anterior motion (SAM) and LVOTO in surgical MV replacement. However, it has been implemented in TMVR to a lesser extent, with few reported cases of LVOTO that lead to conversion to open-heart surgery with controlled cardiac arrest and successful resection of the AML on bypass (63, 64). In these cases, AML resection was a successful bailout option for LVOTO.

### Laceration of the Anterior Mitral Leaflet to Prevent Outflow Obstruction

LVOTO happens mainly due to AML deflection toward the septum, and it can be predicted by measuring the neo-LVOT area or by other anatomical predictors such as acute aortomitral angulation, prominent septal bulge, long AML, and redundant mitral chordae (52, 57, 61, 65). Greenbaum et al. (66) presented case vignettes of LVOTO in support of the removal or reduction of the AML, a technique that has been described in the surgical literature. This led to the development of Laceration of the Anterior Mitral Leaflet to Prevent Outflow ObstructioN (LAMPOON). LAMPOON, the intentional electrosurgical laceration of the AML to prevent LVOTO (67, 68), a challenging procedure, which modified the available surgical approach and has been used successfully before TMVR with the Sapien THV or with dedicated devices designed for the mitral valve, in which successful outcome of patent LVOT was confirmed by measuring LVOT gradient (by means of both echocardiography and catheterization). A National Institutes of Health (NIH)-sponsored trial is ongoing to test the safety and efficacy of LAMPOON in TMVR.

### Alcohol Septal Ablation

Exaggerated basal septal bulge is a risk factor for LVOTO and has been the target of ASA to lower the associated risk. The early cases of ASA in TMVR were performed as a bailout intervention after LVOTO; in these reported cases, patients survived and were hemodynamically stable after the procedure (59, 69, 70). In another study, ASA was performed as a precautionary measure in patients who were identified to be at high risk of LVOTO. Thereafter, ASA has emerged as an intervention to lower the risk of LVOTO by increasing the neo-LVOT surface area by at least 111.2 mm^2^ (interquartile range: 71.4–193.1 mm^2^) and eliminating the exaggerated septal bulge (59). Other techniques such as kissing balloon inflation, medical therapy with aggressive intravenous hydration, and transatrial resection of the AML have been implemented as bailout strategies in LVOTO. However, when outcomes of all the available techniques were compared in the multicenter TMVR registry, survival was achieved only in those who were treated with ASA (33). In the ViMAC study by Guerrero et al. (16), the prevalence of LVOTO was 12% (*N* = 13), with five of the 13 patients treated with medical treatment, one treated with kissing balloon inflation, one treated with surgical intervention, and six patients treated with ASA; among all 13 patients, only two of those who received ASA were alive at 1 year.

## Access-Related Complications

### Transatrial Access

Transatrial TMVR approach with AML resection on cardiac bypass has emerged as an alternative option, especially in patients who are identified at high risk of LVOTO. Moreover, transatrial resection has been employed as a bailout procedure in some of the cases that needed conversion to open-heart surgery and immediate hemodynamic stabilization (16). Praz et al. (22) and Kassar et al. (71) described their experience with 26 consecutive patients with an average STS risk score of 9.4% and 30-days mortality of 27%. In this series, the rate of new-onset atrial fibrillation after the procedure was at 27%. In another single-center case series of six patients who underwent transatrial TMVR for mitral stenosis, 30-days mortality was more than 50% due to severe PVL or device migration (32). In a multicenter study of 21 patients who underwent transatrial TMVR, the authors proposed new techniques of mitral annulus analysis that showed promising transatrial procedural success in patients with severe MAC (72).

### Transapical vs. Transseptal Access

For a successful implantation of THV in TMVR, it is mandatory to achieve robust anchoring of the prosthesis and to overcome the loading force of the left ventricle (14), which is easier through the TA approach compared to the TF approach because of shorter path and coaxiality to the MV (73). Data from the mitral VIVID study showed that most devices were delivered through TA access (79%) (47). In fact, most available dedicated TMVR devices are delivered through TA approach, except for CardiaQ (now Evoque), Cardiovalve, Cephea, and Caisson valves, which are delivered transseptally through TF access. As has already been demonstrated in several TAVR studies, TF is favored over TA, since it is less invasive, associated with less complications, and can be performed under moderate sedation (74–77). Vascular access-related complications can still be seen with TF access. Another complication of the transseptal approach is iatrogenic laceration of the left atrial septum during balloon septostomy or post-procedural expansion of the iatrogenic atrial septal defect (iASD). Therefore, extra caution is mandated when performing balloon sizing of the atrial septum. Data are scarce regarding the outcome of iASD post-procedure and whether it needs to be occluded post-procedure. However, most of the studies point to the fact that most iASDs close at 1 year of follow-up and no correlation with symptoms at 12 months (78, 79). Whereas, TA can be associated with major bleeding, LV apex pseudoaneurysm, and subsequent fibrosis due to sheath positioning. In studies comparing echocardiographic outcomes between TA and TF during TAVR, LVEF recovery and longitudinal strain at follow-up were reduced in the TA group (77). A recent study evaluating mitral ViV TMVR outcomes in a large cohort of 1,529 patients found that the transseptal approach associated with lower 1-year all-cause mortality at 1-year follow-up (16 vs. 22%, *P* = 0.03) (80). In a case series of TMVR comparing TF vs. TA access, there was no difference in procedural duration between the two accesses. However, only TF was associated with an increase in cardiac output and improved survival when compared to TA access (73). Although the Tendyne valve is delivered through TA, data from the first 100 patients showed a procedural success rate of 96% with no intra-procedural mortality (25). Moreover, in a subanalysis of 36 patients who received Tendyne, cardiac CT analysis performed at 1 month post-intervention showed left ventricular end diastolic volumes reverse remodeling. In fact, the authors of this study found that the closer the position of the Tendyne apical pad to the true apex, the more left ventricular remodeling (81). Eleid et al. (44) stratified their TMVR group by early cases of TMVR vs. subsequent cases of TMVR performed after certain modifications applied to TA access led to lower rates of LV perforation and bleeding. However, currently available TMVR devices are mostly delivered through TA access; a significant improvement in the TMVR field will be the implementation and clinical validation of new dedicated TF devices, aiming to minimize access-related complications and to simplify the procedure.

### Left Ventricular Perforation

LV perforation is a rare and fatal complication of MV interventions. In the TMVR studies, LV perforation has been observed especially in the early cases; it is usually related to TA access or directly due to stiff instrumentation when trying to cross the valve, but its rate did not exceed 1% in most studies (49). Moreover, patients at higher risk of LV perforation in transcatheter valvular interventions can be identified on pre-procedural MDCT. For example, in one retrospective study of LV perforations in TAVR, anatomical factors such as small left ventricular cavity, hyperdynamic LV, thick interventricular septum, and narrow mitral angle were predictors of LV perforation (82).

### Left Circumflex Coronary Artery Occlusion

Coronary artery injury is a rare complication of MV surgery with prevalence ranging from 0.5 to 2%. The left circumflex coronary artery (LCx) lies close to the mitral annulus, with the distance ranging from 1 to 9 mm, hence it is at high risk of perioperative injury during MV replacement, and this risk is even higher in left dominant coronary circulation. It can present as an abrupt occlusion intraoperatively or less common as late angina months after the procedure (83). In one study of MDCT analysis of the LCx relation to the mitral annulus, the proximal LCx can be remarkably close to mitral annulus in mid systole. Another study suggests that LCx place can be used as a marker to locate the mitral annulus plane during the procedure, with distance of <5 mm between the two planes (83–87). In TMVR studies, there was no LCx injury or occlusion reported, and rather it seems a complication of transcatheter annuloplasty.

### Conversion to Open-Heart Surgery

Conversion to open-heart surgery is not uncommon; it is usually due to the occurrence of other complications that require immediate surgical intervention to relieve hemodynamic compromise. The decision to convert to open-heart surgery in TMVR is usually made because of LVOTO, valve embolization, severe MR post-deployment, and LV perforation. Conversion to open-heart surgery or LV perforation occurred in 16 patients of the international TMVR registry (3%), with the highest rate in ViMAC, whereas in the ViMAC study by Guerrero et al. (16), Yoon et al. (33), and Kvitting et al. (63), the incidence was 5%, and it was found to be a predictor of all-cause mortality at 1 year.

### Cerebral Embolic Events

The prevalence of cerebrovascular events in the early studies investigating new dedicated TMVR devices seems to be variable, with rates ranging from 0% to as high as 7% (88). However, definitive recommendations on stroke prevention and antithrombotic therapy in TMVR are not well-established. In this context, the well-established clinical experience with surgical mitral valve replacement provides the reference for antithrombotic management in TMVR. Indeed, it has been shown that the risk of embolic cerebrovascular events is higher in the early postoperative period, with overall annual risk of 2.3%, with the highest rates occurring in the first 90 days after surgery (89). This risk is mitigated by the number and duration of the antithrombotic agents prescribed (90, 91). Moreover, patient's related risk factors could increase the risk of cerebral embolic events, such as history of atrial fibrillation, prior embolic events, LV dysfunction, and hypercoagulable states (92, 93). Therefore, the current guidelines recommend the use of oral anticoagulation with vitamin K antagonist (VKA) for 3–6 months (3, 4) at the expense of an increased risk of bleeding.

### Valve Thrombosis

Valve thrombosis can manifest as functional or clinical status deterioration, heart failure symptoms, increased transmitral gradient, or rarely as a visible thrombus or leaflet thickening on echocardiography or MDCT (94, 95). Despite the risk of early valve thrombosis, late valve thrombosis has been recognized as a relevant clinical entity. In a recent study, the median time to explantation for bioprosthetic thrombosis was 2 years, with more than 15% of cases occurring at least 5 years after surgery (96). Hence, long-term clinical and imaging surveillance is indicated to detect delayed valve thrombosis during follow-ups, which can improve with antithrombotic therapy. In the early feasibility studies testing novel THVs in the mitral position, relatively high rates of device thrombosis (6–8%) were reported after Tendyne (Abbott Vascular, Abbot Park, Illinois), Highlife (HighLife Medical, Irvine, California), and Fortis (Edwards Lifesciences, Irvine, California) THV implantation (97). Interestingly, no cases of THV thrombosis were reported after Intrepid THV (Medtronic Inc., Redwood City, California) implantation and the prescription of an aggressive antithrombotic therapy VKA with target international normalized ratio (INR) of 2.5–3.5 plus single antiplatelet therapy for at least 3 months, which came at the expense of higher major bleeding rates (98). Considering the available early evidence, an anticoagulation-based antithrombotic strategy seems to be necessary to prevent the risk of valve thrombosis and thromboembolic events after TMVR, tailoring the intensity and duration of the prescribed antithrombotic regimen on the individual bleeding and thrombotic risk profile of the single treated patient.

### Hemolysis and Paravalvular Leak

PVL is a common complication after prosthetic valve implantation, with a significant increase in morbidity and mortality, especially in patients with severe PVL. The prevalence of PVL in the early studies of surgically implanted bioprosthetic valves was 2.5% (99). After TMVR, the prevalence of PVL that required closure was ~3.5% (33). Moreover, around 3% of patients with PVL after TMVR will develop hemolysis. PVL often results from malposition of the valve or less commonly from valve endocarditis. Mild-to-moderate PVL can frequently be subclinical, with a minimal impact on clinical outcomes, and can be followed up with serial echocardiography studies. Significant (moderate-to-severe) PVL can manifest as heart failure, hemolysis, or a combination of the two (100, 101). Significant PVL is relatively rare in cases of TMVR in non-calcified mitral annuli, while in cases of ViMAC, the rate of moderate-to-severe PVL at 30 days can reach up to 14% (57). Due to the high mortality associated with surgical PVL closure, transcatheter PVL closure emerged as a safe and effective procedure. Today, the operator has the opportunity of using multiple devices concurrently, achieving outstanding results, and eliminating this common and serious complication (102–106).

Other less common fatal structural complications have been reported in TMVR studies with typically early presentation, and the urgent need for conversion to open heart surgery, these include ventricular septal defect, LV pseudoaneurysm, mitral annular disruption, MV leaflet/chordal disruption, and pulmonary vein perforation (34, 36, 49, 107).

## Future Predictions and Implications

**Figure 1:**
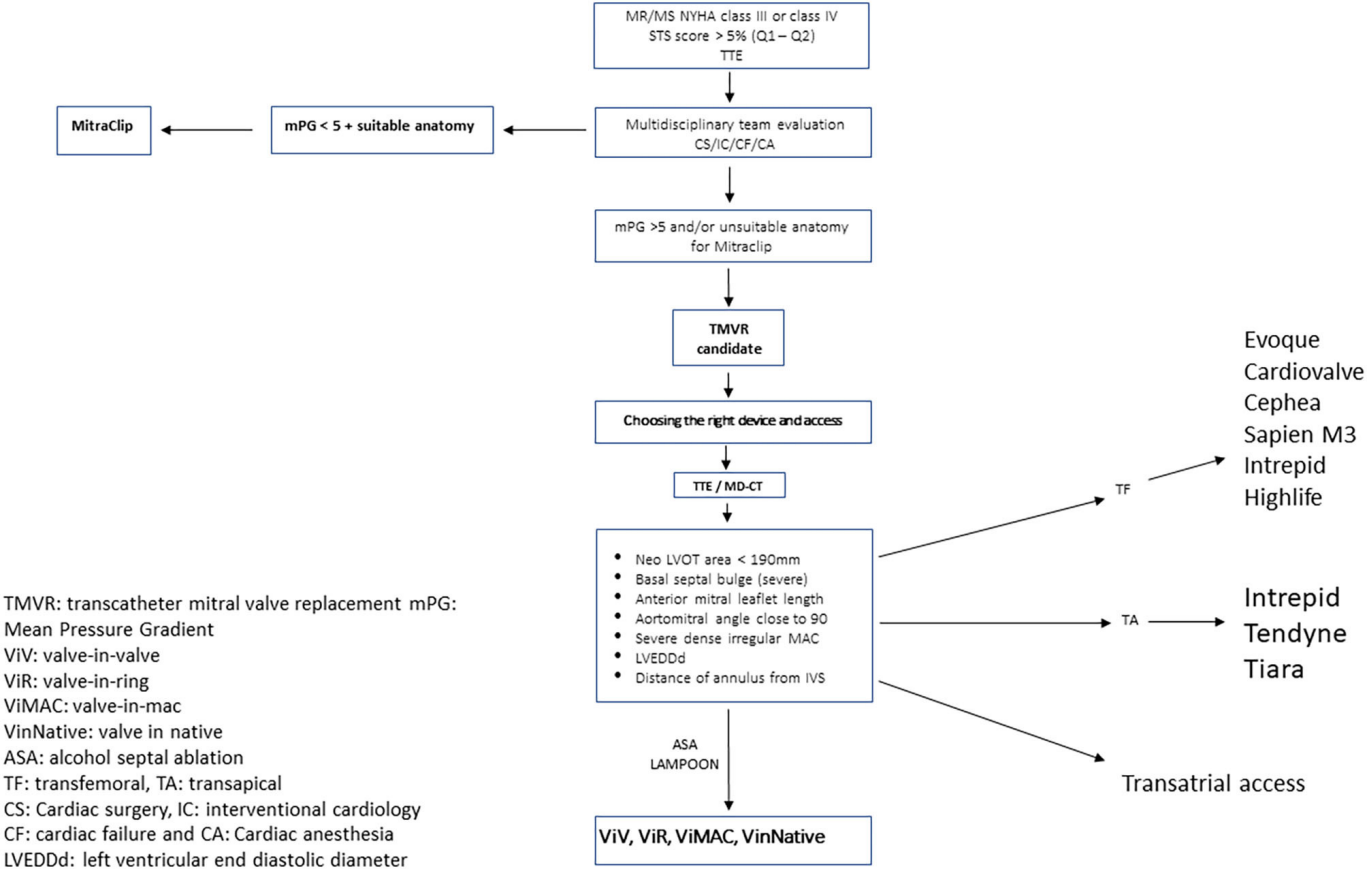
Consort diagram of suggested pre-procedural planning.

As the population ages and the number of patients with MR expand, the therapeutic options available must grow as well. The advent and early success seen with TMVR has yielded much promise. These early outcomes may appear similar to the early success of TAVR, with the majority of patients now receiving transcatheter therapy instead of surgical replacement (108). The TMVR procedure complexity is much higher than that of TAVR and will limit the pace at which operators become comfortable with and regularly incorporate TMVR into their practice. Moreover, patient selection itself is also less defined for TMVR than it is for TAVR. Therefore, one must be cautious in comparing the two therapies due to distinct differences in patient selection, indications, and procedural considerations. Unlike aortic stenosis, for which clear guidelines exist regarding when to intervene, there are currently no clear guidelines as to when a patient should be considered for a mitral intervention for functional MR (4). Hence, we propose a workflow algorithm for the structural heart team when evaluating these patients, given the high morbidity and mortality associated with TMVR; we recommend a heart team comprehensive approach, including interventionalist, echocardiographers, cardiac surgeons, and heart failure specialists, to identify those who would benefit the most from this high-risk intervention. Figure 1 is a brief step-by-step algorithm describing the workflow from identifying those eligible TMVR candidates to post-procedural follow-up (109).

Finally, anatomical considerations such as geometrically dynamic non-planar annular characteristics and complex subvalvular structure make the creation and proper positioning of a valve vastly more difficult at the mitral than the aortic position. The potential for unique complications (i.e., LVOTO), as well as challenging procedural considerations (i.e., transseptal puncture and left atrial maneuvering to achieve coaxial orientation) will require the involvement of not only experienced operators but also a dedicated heart team, surgical and anesthesia staff with every case. These undefined guidelines, unanswered questions, and procedural considerations appear daunting in aggregate (110, 111). Two pivotal ongoing studies will help answer some of these challenging questions regarding different inoperable patient subpopulations with promising devices. The Summit clinical trial has three study cohorts evaluating the Tendyne valve in patients with moderate to severe MR: first cohort is a randomized comparison of the Tendyne heart valve to the MitraClip (Abbott Vascular, Santa Clara, CA), second cohort tests a nonrandomized comparison, and a third cohort evaluates Tendyne device in patients with severe MAC. Another study is the Apollo trial, a prospective non-randomized clinical trial evaluating the safety and efficacy of the Intrepid system (Medtronic, Minneapolis, MN, USA) vs. conventional surgery in patients with severe MR, with another single arm for inoperable patients.

## Conclusion

TMVR represents an evolving therapeutic option to address the unmet clinical need of severe MR. However, several interventional challenges and procedure-related complications need to be addressed. The implementation of multimodality imaging is essential in procedural planning and to identify patients at high risk of complications. Careful pre-procedural planning to help in early identification of those who are at risk for complications, prompt detection and acute management of serious complications, and access refinement are key issues for TMVR advancement.

## Author Contributions

FK performed the literature review, the study outline, and was responsible for manuscript writing. MP and KR participated in the literature review and manuscript writing. EH, MC, YG, and AC helped with writing the manuscript. AL had the overall responsibility for the study and as the corresponding author confirms full access to all aspects of the research and writing process and takes final responsibility for the paper. All authors have participated in the work and have reviewed and agree with the content of the article.

## Conflict of Interest

AL has served on the advisory boards of Medtronic, Abbott Vascular, and Edwards Lifesciences. The remaining authors declare that the research was conducted in the absence of any commercial or financial relationships that could be construed as a potential conflict of interest.

## Abbreviations

MR: mitral regurgitation
MV: mitral valve
MVr: mitral valve repair
TMVR: transcatheter mitral valve replacement
TAVR: transcatheter aortic valve replacement
TA: transapical
TF: transfemoral
LV: left ventricle
MAC: mitral annulus calcification
ViV: valve-in-valve
ViR: valve-in-ring
ViMAC: valve-in-MAC
MDCT: multidetector cardiac tomography
STS: Society of Thoracic Surgeons
THV: transcatheter heart valve
PVL: paravalvular leak
ASA: alcohol septal ablation
LCx: left circumflex coronary artery
AML: anterior mitral leaflet
LVOT: left ventricular outflow tract
LVOTO: left ventricular outflow tract obstruction
iASD: iatrogenic atrial septal defect.
